# Slow Progress Under Brazil’s Native Vegetation Protection Law in the Southeastern Amazon

**DOI:** 10.1007/s00267-026-02454-9

**Published:** 2026-04-21

**Authors:** Hellen Kezia Almada, Rosane Barbosa Lopes Cavalcante, Pedro M. Walfir Souza-Filho, Wilson Nascimento, Sâmia Nunes

**Affiliations:** 1 Instituto Tecnológico Vale, R. Boaventura da Silva, Belém, PA Brazil; 2 Instituto de Pesquisa Ambiental da Amazônia (IPAM), SCLN 211, Asa Norte, Brasília, DF Brazil; 3 Universidade Federal Rural da Amazônia, Tv. Pau Amarelo, Vila Nova, Capitão Poço, PA Brazil; 4 Universidade Federal do Pará, Instituto de Geociências, Av. Augusto Correa 1, Belém, PA Brazil

**Keywords:** Tropical ecosystem, deforestation, remote sensing, forest restoration, rural properties, Brazil environmental policy

## Abstract

There is limited evaluation of the effectiveness of legal environmental protection on private properties in Brazil. This study updates the implementation progress and compensation potential under the Native Vegetation Protection Law (LPVN) in the Itacaiúnas River Basin (BHRI, 41,300 km²), located in Pará, the state with the highest deforestation rates in the Amazon over the past two decades. In 2021, the total Legal Reserve (RL) deficit in BHRI exceeded the surplus by 413 km². A significant portion of this deficit (55% or 2399 km²) could be addressed through compensation, with 3721 km² available within the basin, often in the same or neighboring municipalities. Despite nearly fifteen years since the enactment of the LPVN, progress remains slow in halting deforestation and restoring vegetation. By 2021, 1496 km² of RL deficit should have been under restoration or compensation for medium and large properties. However, only half of the 2008 RL deficit had been addressed. Likewise, just 25% of the 2008 Permanent Preservation Area (APP) deficit had been restored by 2021. Meanwhile, ongoing deforestation in RL and APPs, especially in settlements and small properties, highlights the need for more effective implementation mechanisms. While the state of Pará has made strides in registering and analyzing the Rural Environmental Registry (CAR), implementation of the Environmental Regularization Program (PRA) still faces major challenges. Innovative approaches, such as agroforestry, could help align restoration goals with landowners’ economic interests. Overcoming barriers to regulatory compliance will require coordinated efforts and stronger enforcement mechanisms.

## Introduction

Ecosystem conservation is widely recommended as the primary strategy for maintaining biodiversity, particularly in a context where compensatory mechanisms for biodiversity conservation have expanded globally but may fail to ensure the recovery of ecological functions, species richness, and the conservation of the original species composition affected by land-use change (Bull et al. 2013; Gardner et al. 2013; Gibson et al. 2011; Maron et al. 2025; Penca 2024; Shmelev 2025). However, increasing pressure for economic development has driven a global decline in natural resources, especially in tropical forests where biodiversity is highest. In the Amazon, for instance, approximately 12% (or 44 million hectares) of native vegetation has been lost over the past 40 years, mainly due to pasture expansion (Souza et al. 2020).

In response, governments, companies, and institutions worldwide have pursued actions to mitigate the impacts of deforestation and degradation, contributing to a global race toward net-zero deforestation (MMA 2015; Brazil 2017). Among these measures, forest restoration has emerged as a critical strategy linking environmental conservation, climate resilience, and sustainable development (Aliança pela Restauração na Amazônia 2020). To be effective, however, conservation and restoration strategies must encompass both public and private lands, ensuring forest integrity as a fundamental condition for sustaining essential ecosystem services, including water cycles, water quality, climate regulation, carbon sequestration, and biodiversity conservation (Almada et al. 2024, 2026; Castello et al. 2013; Grimaldi et al. 2014; Nasi et al. 2002). Recent studies further emphasize that the integrity of forest ecosystems underpins the delivery of these services, with disturbances to forest condition substantially reducing their capacity to support ecological processes and ecosystem services (Ali 2023; Almada et al. 2024; Silva et al. 2025).

The conservation and restoration of vegetation on private lands play a central role in national strategies to halt deforestation and recover ecosystem functions. In Brazil, these actions are regulated by the Native Vegetation Protection Law (LPVN, in Portuguese) (Brazil 2012). Brazilian environmental legislation is among the most complex in the world and provides important guidelines for addressing illegal deforestation and promoting vegetation restoration on rural properties (Chiavari and Lopes, 2017; Nunes et al. 2020). To support the implementation of this legislation, specific instruments were established to guide and monitor compliance at the property level, notably the Rural Environmental Registry (CAR, in Portuguese) and the Environmental Regularization Program (PRA, in Portuguese), which aim to promote the environmental adequacy of rural private properties. Under this legal framework, vegetation conservation and restoration are primarily implemented in two types of areas: (i) Legal Reserve areas (RL, in Portuguese), which correspond to a percentage of each rural property that must be maintained under native vegetation and managed for sustainable use and biodiversity conservation; and (ii) Permanent Preservation Areas (APP, in Portuguese), designated to protect environmentally sensitive zones such as riparian vegetation, water springs, and areas with steep slopes (Brazil 2012).

The LPVN introduced differentiated rules based on the timing of deforestation by establishing the concept of consolidated rural areas, defined as areas where native vegetation was suppressed before July 22, 2008. Under this framework, only part of the deforested areas within APPs and RLs are required to be restored (Brazil 2012). This legal arrangement was designed to facilitate the environmental regularization of rural properties, prevent further deforestation in other areas, and ensure access to rural credit. The law also provides differentiated and more flexible restoration requirements for small rural properties. For these properties, restoration obligations in APPs are reduced, while compliance with RL requirements is fulfilled by maintaining the native vegetation existing as of July 22, 2008 (Brazil 2012).

For medium and large rural properties whose RL vegetation areas were smaller than those required by Article 12 of the LPVN prior to July 22, 2008, environmental regularization may occur through restoration or compensation mechanisms (RL deficit eligible for compensation; Article 66), with a 20-year deadline, covering at least one-tenth of the total area to be restored every two years (Brazil 2012). For deforested areas within APPs prior to this date, restoration requirements vary according to property size and waterbody width and must be completed within a maximum period of nine years (Brazil 2012; Pará 2015). In contrast, environmental deficits resulting from illegal deforestation occurring after July 22, 2008, whether in APPs or RLs, are subject to full on-site restoration, with no eligibility for compensation mechanisms (RL deficit to be restored).

However, nearly fifteen years after the enactment of the LPVN, the implementation of the law remains uneven across Brazil. While 20 states have advanced in PRA regulation and implementation, not all have established formal guidelines to address vegetation deficits on private lands. Monitoring of APP and RL deficits also varies substantially among states, with only a limited subset effectively tracking these environmental deficits using high-resolution remote sensing. Among these, the state of Pará stands out as the only one providing public access to up-to-date information and ensuring data transparency (Lopes et al. 2025).

Given this context and considering that the LPVN and PRA established phased restoration timelines, (20 years for RL and up to 9 years for APPs), it is essential to assess how implementation has progressed over time in states where the PRA is more advanced. We therefore present a fine-scale integrated estimate of APP and RL deficits and forest surplus according to land tenure categories for the Itacaiúnas River Basin (BHRI, in Portuguese) in southeastern Brazilian Amazonia. The BHRI offers valuable insights into the complex dynamics of this region, as it encompasses a wide range of land-use and land-cover conditions: approximately one-third of its area is protected by conservation units and Indigenous lands, nearly half of the basin is deforested—primarily due to cattle ranching—and activities such as mining play an important role in the local economy. We address three main questions: (i) what is the status and spatial distribution of LPVN implementation in the BHRI between 2008 and 2021; (ii) to what extent current land-use patterns comply with the minimum legal restoration targets for RL and APP expected by 2021; and (iii) how the 2021 conditions compare with the most recent basin-scale assessment of RL and APP balance available for the same river basin, based on 2017 data (Nunes et al. 2019a).

## Methods

### Study Area

**Fig. 1:**
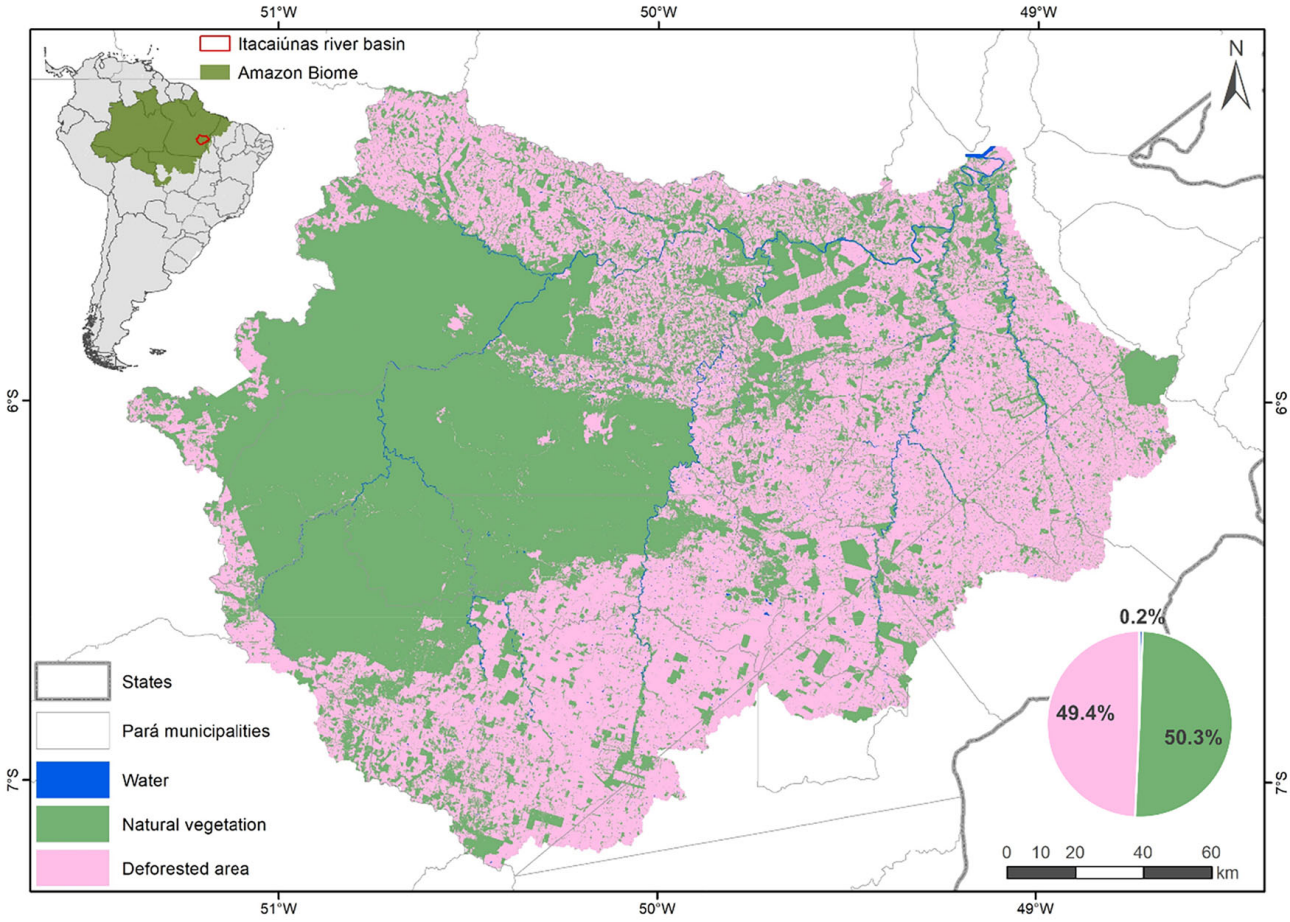
Land-cover map of the Itacaiúnas River Basin in 2021, located in southeastern Pará, Brazilian Amazon. The boundary of the Amazon biome was defined using data provided by the Brazilian Institute of Geography and Statistics (IBGE)

**Fig. 2:**
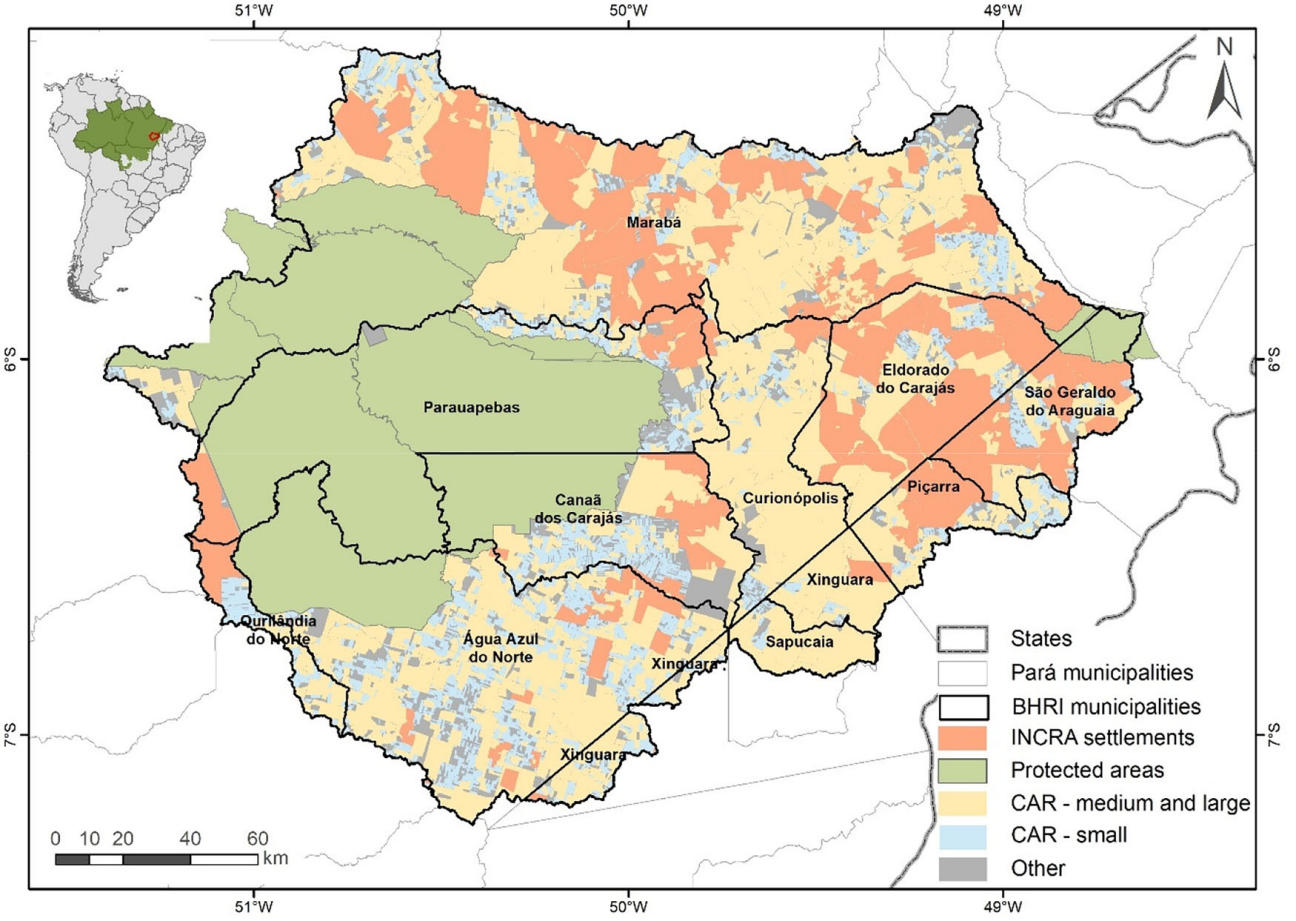
Land occupation of the Itacaiúnas river basin, located in the state of Pará, southeast of the Brazilian Amazon

The BHRI is located in the southeastern Pará, within the Brazilian Amazon, and covers approximately 41,300 km², an area comparable to that of countries such as Switzerland or the Netherlands (Fig. 1). The region encompasses 11 municipalities (Fig. 2). Native vegetation is predominantly tropical forest, and approximately 60% of the remaining native vegetation is contained within a network of protected areas totaling about 12,000 km², composed of indigenous lands and conservation units.

The basin lies within one of Brazil’s most dynamic agricultural frontiers, commonly referred as the “Arc of Deforestation”. Approximately 50% of its area was deforested between the 1970s and the 2020 s, largely driven by the expansion of livestock production (Souza-Filho et al. 2018). The hydrological consequences of land-use change are already evident. Cavalcante et al. (2019) documented significant increasing trends in maximum, mean, and minimum monthly streamflow since the 1970s, associated with land use change. These impacts would likely have been more pronounced in the absence of forest conservation within protected areas (Pontes et al. 2019).

### Data Base

To estimate mandatory forest restoration areas in the BHRI, we combined secondary geospatial datasets, including agrarian reform settlements, protected areas, and the Rural Environmental Registry—CAR, with primary datasets produced by the authors, such as land-cover classification, water bodies, and river networks (Table 1).

**Table 1:** Legal requirements for the restoration of Permanent Preservation Areas (APP), as established by the LPVN, applicable to rural properties

	Criteria	Property size	APP to be restored
Rivers	all	small ( ≤ 1 MF)	5 m
	all	small ( > 1 MF e ≤ 2MF)	8 m
	all	small ( > 2 MF e ≤ 4 MF)	15 m
	≤10 m	medium ( > 4 MF e ≤ 10 MF)	20 m
	>10 m	medium ( > 4 MF e ≤ 10 MF)	^a^30–100 m
	all	medium /large ( > 10 MF)	^a^30–100 m
Springs	–	all	15 m
Lakes and dams	>1 ha	all	15 m
Hilltops	Minimum height of 100 m and slope >25°	all	all^a^
Slope	>45°	all	all^a^

These requirements apply to APPs deforested up to July 22, 2008

^a^The LPVN imposes a restoration obligation only for areas deforested after 22 July 2008

### BHRI Land Tenure Situation

To assess the need for forest restoration and/or compensation of forest deficit in the basin, we produced a land-cover map of the study region comprising four main classes (Fig. 2): (i) CAR registered properties classified by property size: small (up to 4 fiscal modules [MF]), medium (greater than 4 and up to 15 MF), and large (greater than 15 MF) (Brazil 1993); (ii) agrarian reform settlements, defined as groups of small rural properties established by the National Institute for Colonization and Agrarian Reform (INCRA) for low-income families; (iii) protected areas, including conservation units and indigenous lands; and (iv) other areas, encompassing urban areas, private properties not registered in the CAR, and undesignated public lands. Land-tenure categories directly determine the legal requirements for forest protection, restoration, and compensation under the LPVN.

### Land Cover Classification

Land cover classification for the Itacaiúnas River Basin was conducted for four reference years: 2008, 2013, 2017, and 2021. These years were used as temporal benchmarks to reconstruct the land-cover history of the basin, capturing key stages of land-cover change and allowing the identification of deforestation timing in relation to the legal milestones established by the LPVN.

Land-cover maps of the basin were developed using image mosaics from the Landsat-5, Landsat-8, and Sentinel-2A satellites (Table 1), using a multitemporal object-based image analysis (GEOBIA) approach (Blaschke 2010; Souza-Filho et al. 2016). Image classification and digital image processing followed the methodology proposed by Souza-Filho et al. (2016, 2018). The land cover classes considered were native vegetation (henceforth referred to as forest, due to the predominance of forest formation, although including patches of native rupestrian fields in mountainous areas), deforested areas (predominantly pasture, but also including mining, urban, and agricultural areas), and water bodies. Areas were considered to have progressed toward restoration when forest cover was detected in 2021 in locations previously classified as deforested in 2008, without inferring vegetation condition or successional stage.

To assess the temporal consistency of our classification results, we compared them with the MapBiomas Collection 7 database (Souza et al. 2020). This comparison was conducted to verify whether the land-cover changes observed over time were attributable to actual changes in land cover or potentially influenced by shifts in the spatial resolution and satellite imagery sources used in our classification across different years. Forest and deforested classes from our classification were plotted alongside MapBiomas classifications for the corresponding reference years (2008, 2013, 2017, and 2021). Both datasets exhibited similar temporal trends in forest loss and deforestation, despite differences in the absolute magnitude of class areas resulting from differences in spatial resolution and classification methods. Detailed procedures and additional results are provided in the Supplementary Material (Section 1).

### Analysis of the BHRI Legal Reserve (RL) and Permanent Preservation Areas (APP)

For the RL analysis, we evaluated 6352 properties, including 6233 rural properties registered in the CAR and 119 agrarian reform settlements. Protected areas were excluded from the analysis, and overlaps between rural properties were addressed following the procedures described by Nunes et al. (2016).

RL requirements and deficit categories were defined according to the LPVN (Brazil 2012). In the Brazilian Amazon, the standard RL requirement corresponds to 80% of the property area. However, Article 12 of the LPVN establishes specific provisions for consolidated areas, allowing, under certain conditions, a reduction of the RL requirement down to 50% for medium and large properties (Brazil 2012). These conditions depend on property size, the existence of ecological–economic zoning, the proportion of protected areas within the municipality, and the timing of deforestation. For small rural properties, RL compliance corresponds to maintaining the native vegetation existing as of July 22, 2008; the same rule applies to agrarian reform settlements (Brazil 2012).

Based on land-cover conditions observed in 2021 and the applicable legal thresholds, we classified RL status at the property level into four categories: (i) RL deficit eligible for compensation, corresponding to illegal deforestation prior to 2008; (ii) RL deficit to be restored, corresponding to illegal deforestation after 2008; (iii) deforestable surplus, corresponding to forest cover that exceeds the legally required RL percentage and may therefore be legally cleared; and (iv) compensation-only surplus, defined as vegetation within RL that may be used to compensate off-site deficits but is not legally eligible for deforestation.

The compensation-only surplus comprises two situations under the LPVN: (a) on medium and large properties, forest cover located between the reduced RL threshold (e.g., down to 50%) and the standard RL requirement (80%), which cannot be legally cleared but may be allocated for compensation; and (b) on small rural properties and agrarian reform settlements, the entire RL area, which may be fully used for compensation purposes (Brazil 2012).

For this study, we considered Permanent Preservation Areas (APPs) along rivers, lakes, reservoirs, water springs, hilltops, and areas with slopes greater than 45° across the entire basin, including both protected and private lands, in accordance with the LPVN (Brazil 2012). APP boundaries were spatially delimited following the methodology proposed by Nunes et al. (2019a).

APPs were classified into four categories: (i) total APP, corresponding to the full extent of legally designated APPs; (ii) APP with forest, comprising areas under native vegetation cover; (iii) APP to be restored, corresponding to minimum restoration areas required by law, including APPs deforested after July 22, 2008, in accordance with Article 61-A (Brazil 2012) (Table 1); and (iv) consolidated APP, defined as deforested areas within APPs where restoration is not legally mandatory under the LPVN.

### Reconstruction of the 2008 Legal Baseline

To evaluate compliance with the LPVN, we reconstructed RL and APP deficits for the year 2008, which represents the legal baseline established by the law. This was done by overlaying CAR and agrarian reform settlement boundaries with the 2008 land cover map (Table 2).

**Table 2:** Spatial datasets used for land cover classification and environmental analyses in the Itacaiúnas River Basin, including data source, reference year, spatial resolution or scale, and satellite imagery

Database	Source	Year	Scale/Spatial Resolution	Satellite image
Settlements	INCRA^a^	2013	–	–
Protected areas	ICMBio^b^	2015	–	–
CAR	SEMAS-Pará^c^	2021	–	–
Land Cover Classification		2008, 2013	1:150.000 (30 m)	Landsat-5, Landsat-8
Land Cover Classification		2017, 2021	1:50.000 (10 m)	Sentinel-2A
Water bodies		2021	1:50.000 (10 m)	Sentinel-2A
Rivers^d^		2011	1:50.000 (12.5 m)	MDE-Alos Palsar
APPs^d^		2011	1:50.000 (12.5 m)	MDE-Alos Palsar

*CAR* Rural Environmental Registry, *MDE* Digital Elevation Model, *ITV* Vale Technological Institute

^a^National Institute of Colonization and Agrarian Reform

^b^Chico Mendes Biodiversity Institute

^c^Secretary of State for the Environment and Sustainability—Pará State

^d^Nunes et al. (2019a)

In accordance with the LPVN, RL deficits prior to 2008 in small rural properties and agrarian reform settlements were considered legally amnestied. However, these properties may still present restoration obligations resulting from deforestation occurring after 2008.

RL deficits were classified according to the timing of deforestation. Deficits resulting from deforestation prior to July 22, 2008, may be regularized through on-site restoration, assisted natural regeneration, or off-site compensation within the same biome. In contrast, deforestation occurring after July 22, 2008, generates RL deficits that are not eligible for compensation and must be fully restored on-site.

### Assessment of LPVN Implementation Over Time

To assess the implementation of the LPVN, we compared the reconstructed 2008 baseline with land-cover conditions observed in 2021 under the restoration timelines established by the law. According to the LPVN and its regulatory instruments, restoration actions should have begun within two years after the law’s enactment in 2012, with at least one-tenth of the total RL deficit regularized every two years, and full compliance achieved within 20 years for RL and nine years for APP (Brazil 2012; Pará 2015).

Accordingly, by 2021, minimum legal compliance corresponds to the resolution of approximately 50% of the RL deficit existing in 2008. We therefore estimated the cumulative RL deficit expected to be regularized through restoration or compensation in 2014, 2016, 2018, 2020, and 2022 by applying successive 10% reductions to the pre-2008 deficit.

Medium and large properties were classified according to whether they had restored at least 50% of their pre-2008 RL deficit by 2021 or less than this threshold. For each group, we quantified: (a) the area of RL deficit restored on-site; and (b) the area of new RL deficit generated after 2008, corresponding to illegal deforestation subject to on-site restoration. This allowed us to evaluate both compliance with restoration targets and the occurrence of additional deforestation, including among properties meeting the minimum legal threshold.

Finally, we contrasted the 2021 results with basin-scale estimates from 2017 reported by Nunes et al. (2019a). To ensure comparability, this analysis was restricted to the same set of CAR registrations used in the 2017 assessment.

## Results

### Land Tenure and Land Occupation in the BHRI

The BHRI is predominantly occupied by rural properties registered in the CAR, which together account for 64% (26,239 km²) of the basin area. Of this total, 10% (4058 km²) correspond to small rural properties, 11% (4442 km²) to medium-sized properties, 23% (9927 km²) to large properties, and 20% (8148 km²) to agrarian reform settlements. Protected areas represent 29% (12,012 km²) of the basin, while areas under other land tenure categories (urban areas, private properties not registered in the CAR, and undesignated public lands) account for the remaining 7% (3054 km²) (Fig. 2).

### Legal reserve and Legal Reserve deficits and surpluses

**Fig. 3:**
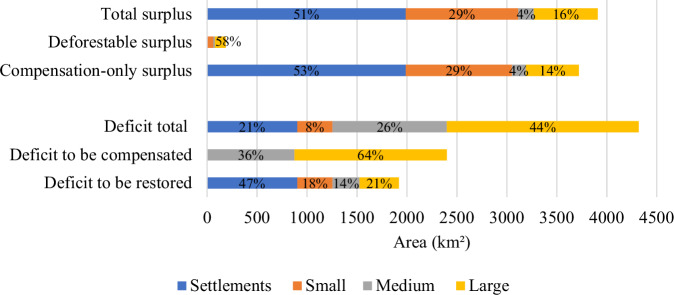
Estimates of Legal Reserve deficits and surpluses in CAR-registered rural properties and INCRA agrarian reform settlements, for the year 2021 in the Itacaiúnas River Basin, southeastern Brazilian Amazon. Percentages refer to the total Legal Reserve deficits and surpluses within the basin

In 2021, the total RL deficit estimated for the BHRI was 4321 km², corresponding to 21% of the basin’s deforested area and 10.5% of the total basin area. Of this total, 55% (2399 km²) corresponds to RL deficits eligible for regularization through restoration or compensation, associated with deforestation that occurred prior to July 22, 2008. The remaining 45% (1922 km²) corresponds to RL deficits subject to mandatory on-site restoration, resulting from deforestation after this date (Fig. 3).

Large properties accounted for 64% of the total compensable RL deficit area, while medium-sized properties accounted for the remaining 36%. When normalized by the required RL area, medium-sized properties exhibited a higher average relative compensable deficit (40.7%) than large properties (30.8%). Agrarian reform settlements and small properties did not present compensable RL deficits, as their RL requirement is legally limited to the native vegetation existing as of July 22, 2008.

RL deficits subject to mandatory on-site restoration were unevenly distributed across land tenure categories. Agrarian reform settlements concentrated 47% of the total restoration demand, followed by large properties (21%), small properties (18%), and medium-sized properties (14%) (Fig. 3). When normalized by the required RL area, agrarian reform settlements and small properties showed the highest average relative restoration deficits, with mean unmet RL values of 45.6% and 46.5%, respectively. Medium-sized properties presented a lower average relative deficit (11.9%), while large properties exhibited the lowest relative deficit (7.9%).

The total RL forest surplus mapped in the basin was 3908 km². Of this total, 95% (3721 km²) corresponds to compensation-only surplus, while only 5% (187 km²) corresponds to deforestable surplus. Deforestable surplus was predominantly located in large properties (58%), followed by small properties (34%) and medium-sized properties (8%). Agrarian reform settlements did not present deforestable surplus. In contrast, compensation-only surplus was mainly concentrated in agrarian reform settlements (53%), followed by small properties (29%), large properties (14%), and medium-sized properties (4%) (Fig. 3).

At the basin scale, total RL deficits exceed total RL surpluses by 413 km². Nevertheless, the compensation-only surplus available within the basin is sufficient to offset all RL deficits eligible for compensation, frequently within the same municipality or in neighboring municipalities (Supplementary Material, Section 2).

### Implementation Status of RL Obligations

**Fig. 4:**
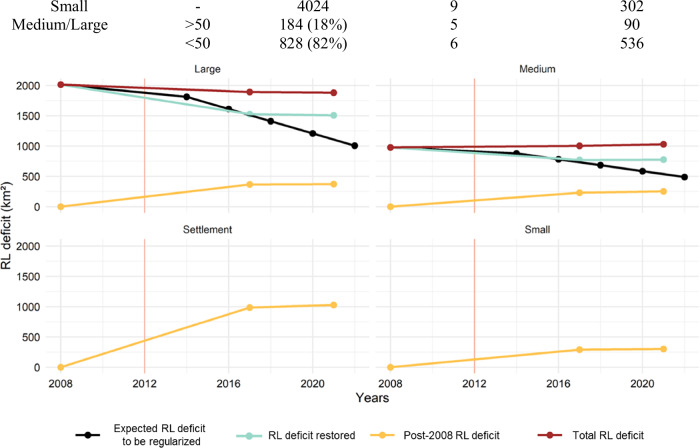
Temporal evolution of RL deficits in the Itacaiúnas River Basin, southeastern Brazilian Amazon. Lines represent: Expected RL deficit to be regularized (black), corresponding to the remaining pre-2008 RL deficit assuming compliance with the LPVN restoration timeline; RL deficit restored (green), representing areas where forest cover was detected in 2021 in locations previously classified as deforested in 2008; RL deficit due to post-2008 deforestation (yellow), corresponding to new illegal deforestation subject to mandatory on-site restoration; and Total RL deficit (red), defined as the sum of the remaining pre-2008 deficit and post-2008 deforestation. The vertical red line indicates the enactment of the LPVN in 2012. Estimates for 2017 were derived from Nunes et al. (2019a), while values for 2008 and 2021 were calculated in this study, using consistent spatial units and property boundaries

In 2008, the RL deficit in the BHRI totaled 2992 km², with 67% located in large properties and 33% in medium-sized properties. According to the implementation timeline established by the LPVN, approximately 50% of this deficit should have been regularized by 2022. However, by 2021 only 707 km² (24% of the expected area) exhibited forest cover in locations previously classified as deforested within the properties where the deficit originated (Fig. 4). Of this restored area, 72% (507 km²) occurred in large properties and 28% (200 km²) in medium-sized properties. Fewer than 20% of medium and large properties restored 50% or more of their RL deficit eligible for restoration or compensation (Table 3).

**Table 3:** Distribution of RL restoration outcomes and remaining restoration obligations by 2021 in rural properties registered in the CAR and agrarian reform settlements in the Itacaiúnas River Basin, southeastern Brazilian Amazon

	Restored (%)	Number of properties	Mean area of deficit to be restored (%)	Total area to be restored (km²)
Settlement	–	119	14	1029
Small	–	4024	9	302
Medium/Large	>50	184 (18%)	5	90
	<50	828 (82%)	6	536

Restored (%) refers to the proportion of the pre-2008 RL deficit restored within the property where the deficit originated. Mean area of deficit to be restored (%) and Total area to be restored (km²) correspond to RL deficits resulting from deforestation after July 22, 2008, which are subject to mandatory on-site restoration RL deficits prior to 2008 in small rural properties and agrarian reform settlements were legally amnestied and therefore are not included in the restoration percentage

**Fig. 5:**
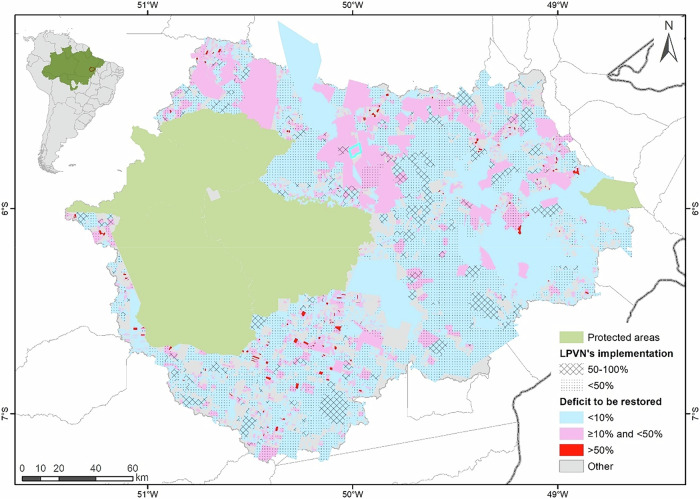
LPVN implementation status and Legal Reserve (RL) deficits subject to restoration in CAR-registered rural properties and INCRA agrarian reform settlements as of 2021, in the Itacaiúnas River Basin, southeastern Brazilian Amazon

Between 2008 and 2021, total deforestation in the BHRI increased by 2578 km² (6%), while the area subject to mandatory restoration due to post-2008 illegal deforestation reached 1957 km²—substantially exceeding the area restored during the same period (707 km²). Restoration, however, does not necessarily mean progress in environmental compliance, since it may have occurred in areas outside the legal reserve. Even among properties that restored at least 50% of their pre-2008 RL deficit, additional deforestation after 2008 was observed, although at lower rates than in properties that did not reach this threshold (Fig. 5; Table 3). The area subject to mandatory restoration due to post-2008 deforestation was proportionally higher in agrarian reform settlements and small properties.

### Permanent Preservation Areas

**Fig. 6:**
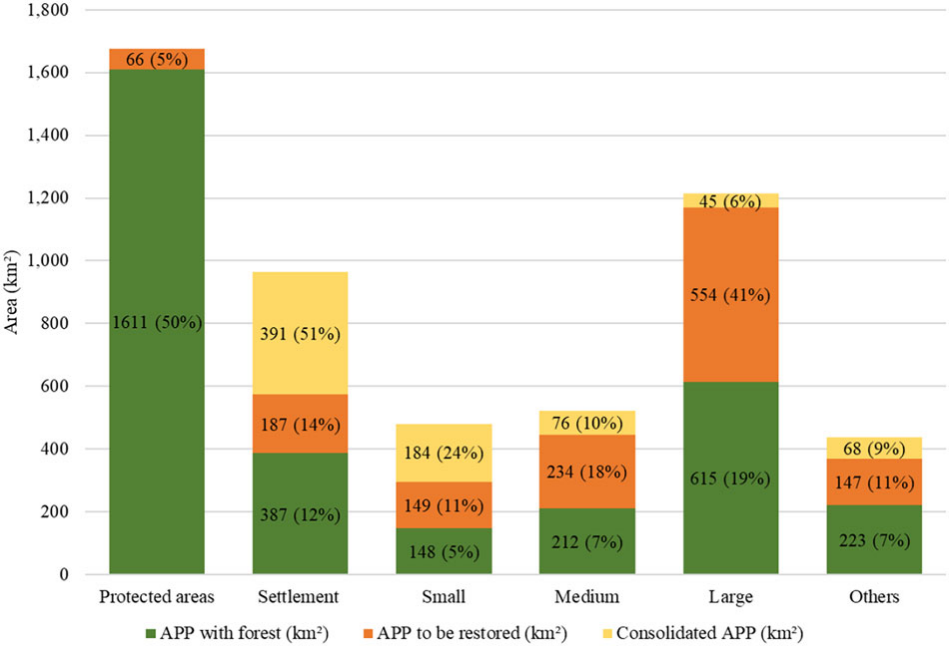
Distribution of Permanent Preservation Areas (APPs) with forest cover, APPs to be restored, and consolidated APPs across protected areas, private properties, and undesignated lands in the Itacaiúnas River Basin, southeastern Brazilian Amazon. Percentages represent the relative distribution of APP categories within each land-tenure class

Based on land cover conditions observed in 2021, the total area classified as APPs in the BHRI was estimated at 5248 km². Of this total, approximately 60% (3163 km²) remained under native vegetation, 25% (1316 km²) corresponded to APPs requiring restoration under the LPVN, and 15% (762 km²) consisted of consolidated APPs, where restoration is not legally required (Fig. 6).

Forested APPs were predominantly located within protected areas, which accounted for approximately half of all APPs with remaining vegetation in the basin. Consolidated APPs were broadly distributed across the deforested portion of the basin, whereas APPs requiring restoration were spatially concentrated in isolated areas associated with more recent deforestation events occurring after July 22, 2008. The municipal-level distribution of APPs with forest cover and APPs requiring restoration is presented in the Supplementary Material (Section 3).

### APP Deficits and LPVN Implementation Over Time (2008–2021)

To assess the implementation of the LPVN, APP conditions observed in 2021 were compared with the legal baseline established in 2008. In 2008, the total APP deficit in the BHRI was 1348 km². According to the PRA-PA, all APP deficits should have been fully restored within nine years of the program’s publication (Pará 2015). However, by 2021 only 347 km² of this deficit had been restored, corresponding to 26% of the APP deficit identified in 2008.

**Fig. 7:**
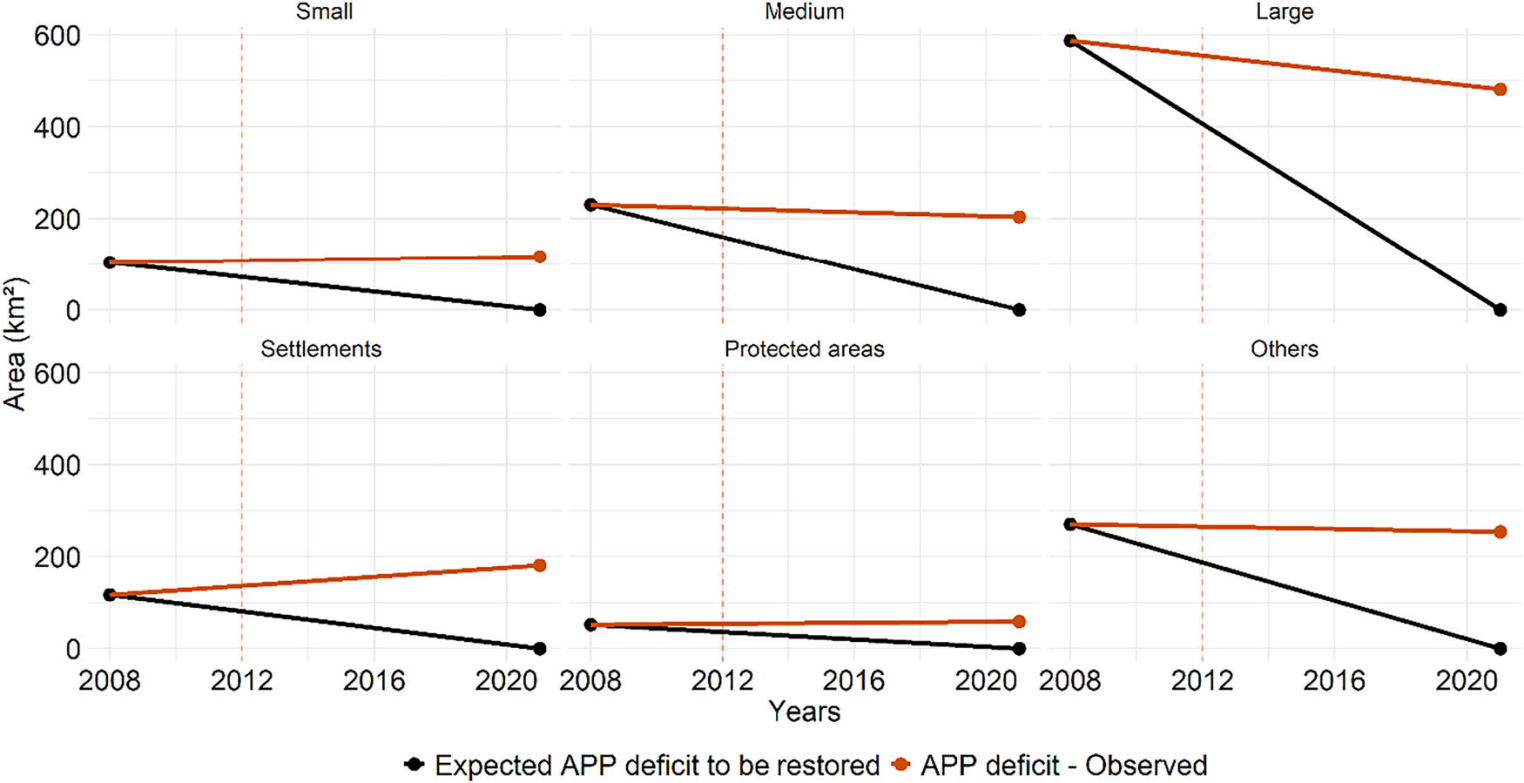
Temporal evolution of Permanent Preservation Area (APP) deficits by land-tenure category in the Itacaiúnas River Basin, southeastern Brazilian Amazon. The orange line represents the observed APP deficit, corresponding to APP areas without detectable forest cover. The black line represents the expected APP deficit to be restored under legal compliance with the LPVN. The dashed red vertical line indicates the enactment of the LPVN in 2012

Over the same period, an additional 281 km² of APPs were deforested after July 22, 2008, representing approximately 5% of the total APP area in the basin. As a result, the total APP area requiring restoration in 2021 amounted to 1295 km², a value very similar to that observed in 2008 (Figs. 6 and 7). In absolute terms, restoration gains since 2008 were therefore largely offset by post-2008 deforestation, resulting in minimal net change in the APP restoration liability over time.

When disaggregated by land tenure category, relative APP restoration rates were similar across protected areas, agrarian reform settlements, and large rural properties, each reaching approximately 27% of their respective baseline deficits. In absolute terms, however, restoration gains were uneven. Large rural properties accounted for the largest restored area (156 km²), followed by agrarian reform settlements (32 km²) and protected areas (14 km²).

In contrast, post-2008 deforestation within APPs showed marked differences across land tenure categories. Agrarian reform settlements exhibited the highest relative proportion of APP deforestation after 2008 (10%), followed by small properties (9%), areas without CAR registration (8%), medium-sized properties (6%), and large properties (5%) (Fig. 6). In absolute terms, settlements also concentrated the largest area of post-2008 APP deforestation (96 km²), followed by areas without CAR registration (54 km²) and large rural properties (51 km²).

**Fig. 8:**
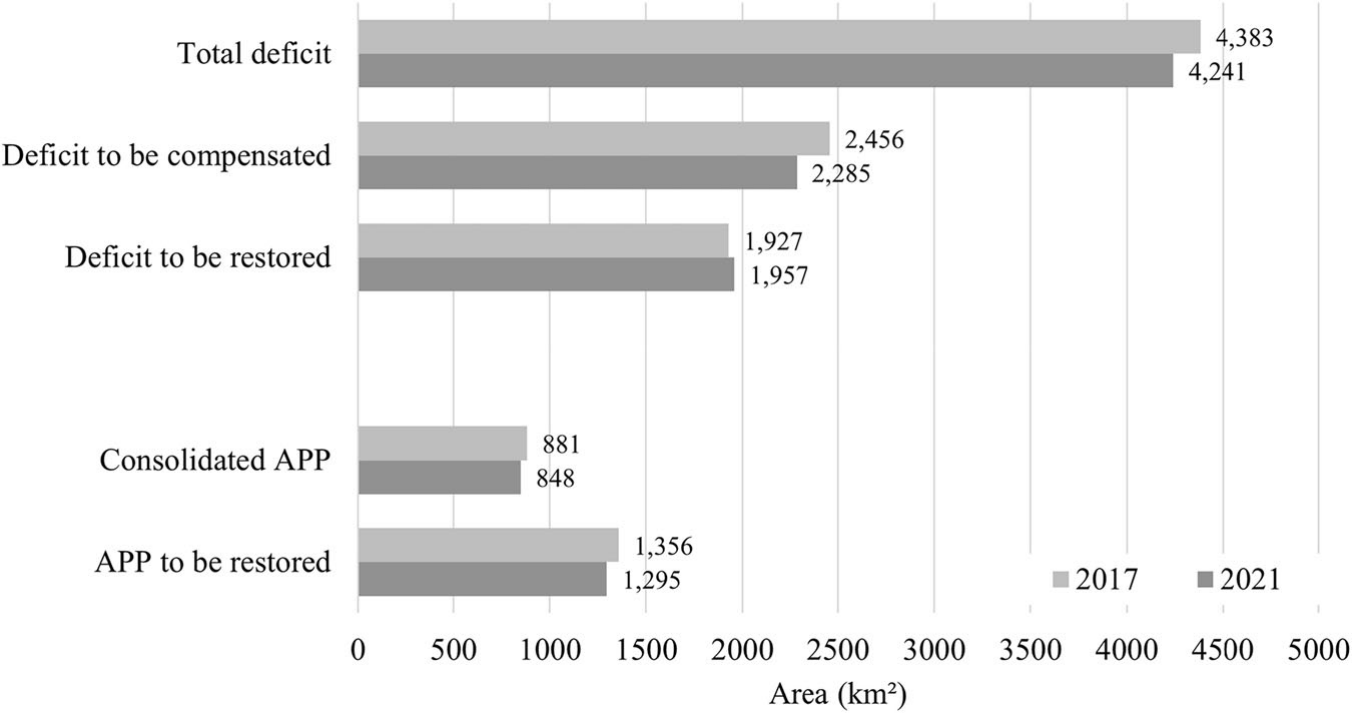
Comparison of Legal Reserve (RL) and Permanent Preservation Area (APP) deficits and surpluses between 2017 and 2021 for CAR-registered rural properties (as of 2017) and INCRA agrarian reform settlements in the Itacaiúnas River Basin, state of Pará, southeastern Brazilian Amazon

We also compared the 2021 results with data from 2017 reported by Nunes et al. (2019a). Overall, RL and APP indicators showed limited variation between 2017 and 2021 (Fig. 8). During this period, the total RL deficit decreased by 142 km². This reduction was driven by a decrease of 172 km² in RL deficits eligible for compensation. In contrast, RL deficits subject to mandatory on-site restoration increased by 30 km² between 2017 and 2021.

For APPs, the area requiring restoration declined by 61 km² (4%) between 2017 and 2021, decreasing from 1356 to 1295 km² (Fig. 8). Consolidated APP areas also declined slightly over the same period, from 881 km² in 2017 to 848 km² in 2021, corresponding to a reduction of 33 km² (4%).

## Discussion

Despite nearly fifteen years since the enactment of the LPVN, our results reveal a substantial gap between legal expectations and on-the-ground outcomes in the BHRI. This imbalance highlights a structural weakness in LPVN implementation: while the law establishes restoration obligations, it has been far less effective in preventing new deforestation. The results show that forest restoration progress was much more slow than the pace of new clearing, leading to stagnation—or even regression—in environmental compliance. Considering the baseline of 2008, the total RL deficit in 2021 was 44% greater, reaching 4321 km², despite the existence of clear legal timelines requiring progressive resolution of pre-2008 deficits and full restoration of post-2008 illegal deforestation. By 2021, only 707 km² of RL deficit had been restored on-site, representing less than half of what would be expected under a linear compliance trajectory and falling far short of the extent of post-2008 illegal deforestation that generated new mandatory restoration obligations (1957 km²). The comparison with basin-scale estimates from 2017 provides additional evidence that restoration efforts have stagnated in recent years. Between 2017 and 2021, reductions in RL deficits were driven exclusively by decreases in deficits prior to 2008, while deficits requiring mandatory restoration increased.

Similar patterns have been documented across the Brazilian Amazon, where restoration commitments have advanced more slowly than land-use conversion, particularly in periods of weakened environmental governance (Ferrante & Fearnside, 2019; Souza et al. 2020; Rajão et al. 2020). Although landowners in eastern Amazonia have increasingly registered their properties, CAR registration had only a temporary effect on reducing deforestation among smallholders, with this effect quickly fading over time (L’Roe et al. 2016; Azevedo et al. 2017). This finding aligns with evidence that restoration mandates, when not coupled with strong enforcement and monitoring, often fail to offset ongoing habitat loss (Maron et al. 2025; Strassburg et al. 2022, Min et al. 2025). The expansion of market mechanisms has been proposed to complement government policies to encourage the end of deforestation and forest restoration (Azevedo et al. 2017; Rodrigues et al. 2025).

Despite the change in legislation that allowed for the reduction of the legal reserve area to be restored in these properties, our analysis demonstrates that the mandatory restoration after 2008 is concentrated in agrarian reform settlements and small rural properties, which together account for the highest relative restoration deficits. In contrast, medium and large properties concentrate most of the RL deficit eligible for compensation but exhibit lower relative deficits when normalized by legally required RL area. Similar land tenure-dependent patterns have been observed in other Amazonian regions, where settlements and smallholders face greater challenges in preventing new deforestation due to limited access to credit, technical assistance, and enforcement presence (Lopes et al. 2023; Pacheco et al. 2021). However, this is not a reality in the Amazon in general; in other regions, large and medium-sized properties may be the main contributors to the deficit. Longer land tenure and transitions to cattle grazing, but not agricultural rents (Schons et al. 2019), and pervasive conditions of social stress combined with lived experiences of contradictory legal processes are also associated with non-compliance, reinforcing structures of social exclusion (Schmidt et al. 2014). These results are aligned with broader findings that restoration policies often impose higher relative costs on vulnerable landholders, unless accompanied by targeted support mechanisms (Erbaugh et al. 2020; Tedesco et al. 2025). Therefore, that is a need for targeted technical assistance, livelihood alternatives, and monitoring strategies that address the specific vulnerabilities of these areas.

Although the compensation-only surplus identified in the BHRI is sufficient to offset all RL deficits eligible for compensation under the LPVN, this result requires careful legal and ecological interpretation. This surplus does not represent vegetation that can be legally cleared; rather, it reflects a regulatory surplus created by LPVN provisions that restrict legal deforestation while allowing specific portions of vegetation to be used for compensation purposes (Stabile et al. 2022). For medium and large properties, compensation-only surplus corresponds to vegetation located between 50% and 80% of the property area—that is, the portion of the RL that exceeds the minimum restoration requirement but remains legally protected from conversion. In small properties and agrarian reform settlements, the entire RL area may be used for compensation purposes. However, vegetation that regenerated after July 22, 2008 is not specifically prioritized within compensation schemes, as it may be legally cleared under certain conditions and therefore does not constitute a permanently protected component of the RL.

As a result, the compensation-only surplus corresponds to vegetation that is already legally protected and eligible exclusively for use in compensation schemes, rather than representing additional conservation beyond existing legal requirements. In the BHRI, this compensation-only surplus is predominantly concentrated in small properties and agrarian reform settlements, where compensation mechanisms may primarily formalize legal compliance rather than generate additional conservation outcomes. In such contexts, compensation transactions do not necessarily prevent future deforestation or promote ecological recovery beyond existing legal requirements, raising important concerns regarding ecological additionality (Bull et al. 2013; Maron et al. 2015).

This tension between legal compliance and ecological effectiveness has been widely discussed in international literature on biodiversity offsets and compensation schemes. A growing body of evidence shows that compensation mechanisms often fail to ensure equivalence in ecological functions, species richness, and species composition, particularly when they rely on pre-existing habitats rather than on additional conservation or restoration actions (Bull et al. 2013; Maron et al. 2025; Abdo et al. 2021; Borges-Matos et al. 2025; Penca, 2024; Shmelev, 2025). In this context, restoration and natural regeneration are widely recognized as more effective pathways for biodiversity recovery and the maintenance of local ecological processes than compensation alone. At the same time, our results highlight a critical opportunity for policy design: the fraction of surplus that remains legally deforestable represents the portion where real environmental additionality can still be achieved. Prioritizing incentives and protective mechanisms to conserve this deforestable surplus could prevent future forest conversion and generate tangible conservation gains on private lands that are not otherwise fully protected by the LPVN (Nunes et al. 2019a; Stabile et al. 2022).

In this context, emerging approaches that explicitly recognize the intrinsic value of standing forests, due to their provision of ecosystem services and biodiversity maintenance, offer promising pathways to complement traditional command-and-control instruments. Initiatives such as the CONSERV concept proposed by Stabile et al. (2022) exemplify how voluntary, market-based mechanisms can transform conserved native vegetation into a viable revenue stream for rural landowners, particularly in areas not legally protected. By being voluntary, relatively simple, and less bureaucratic than public environmental programs, such mechanisms may increase landowner engagement and reduce incentives for future deforestation.

When strategically combined with restoration obligations and compensation rules under the LPVN, these approaches can enhance ecological additionality by safeguarding forests that would otherwise remain vulnerable to legal conversion, while simultaneously addressing socio-economic constraints faced by landowners. Similar strategies have been identified as cost-effective pathways to strengthen conservation outcomes and avoid symbolic compliance or greenwashing in forest compensation systems in Brazil and other tropical regions (Strassburg et al. 2019; Erbaugh et al. 2020; Borges-Matos et al. 2025; Min et al. 2025).

Compensation may nonetheless play an important socio-environmental role, especially in landscapes dominated by smallholders and agrarian reform settlements. In these contexts, compensation mechanisms can provide income, reduce incentives for illegal clearing, and support vulnerable rural populations, even when ecological gains are limited. This dual role reflects broader global debates on how to balance conservation effectiveness, equity, and livelihoods in restoration and offset policies (Bull et al. 2013; Tedesco et al.^ 2025^; Min et al. 2025). However, realizing these potential social benefits requires substantially greater transparency and traceability in the implementation of compensation. At present, publicly available data does not allow systematic identification of which properties with RL deficits are engaging in compensation mechanisms, where compensation is occurring, or whether transactions effectively reduce pressure on forests. Similar limitations in data transparency and monitoring have been identified as critical barriers to evaluating the effectiveness of offset and compensation schemes in other regions (Abdo et al. 2021; Maron et al. 2025; Penca 2024). Strengthening public registries, integrating compensation information into land-use monitoring systems, and ensuring open access to compliance data are therefore essential to assess both the ecological and social outcomes of compensation under the LPVN.

APP restoration followed a pattern similar to that observed for RL, with only marginal reductions in restoration demand between 2008, 2017, and 2021. Agrarian reform settlements tend to concentrate higher relative rates of riparian deforestation, whereas large properties account for most of the absolute extent of APP clearing. This pattern is consistent with findings from Mato Grosso, where the area newly cleared within APPs has been comparable to the area restored, resulting in limited net reductions in APP deficits (Preto et al. 2022). Although the implementation of the LPVN contributed to a general reduction in riparian vegetation deficits associated with pre-2008 deforestation, it has proven insufficient to halt new deforestation within APPs.

The persistence of APP degradation is particularly concerning given the critical role of riparian vegetation in hydrological regulation, erosion control, and biodiversity conservation (Almada et al. 2019; Biggs et al. 2019; Vagheei & Boano et al. 2025). Moreover, deforestation within APPs has been shown to exceed deforestation outside these areas in most municipalities of Pará, highlighting persistent enforcement failures (Nunes et al. 2019b). Differences in landowner awareness may partly explain this pattern, as compliance efforts tend to focus more strongly on RL obligations than on APP use restrictions (Schmidt and McDermott 2014).

Our results also show that approximately 15% of APPs in the basin are classified as consolidated and therefore not subject to mandatory restoration, with agrarian reform settlements and small properties concentrating on a large share of these areas. Although the consolidated regime was introduced to facilitate legal regularization and reduce social inequities, it appears to have favored areas with historically high levels of non-compliance while delivering limited ecological recovery (Brancalion et al. 2016). The relaxation of restoration requirements reduced formal obligations but did not translate into substantial vegetation recovery and may have reinforced perceptions of impunity under weak enforcement conditions (Ferrante and Fearnside 2019; Rajão et al. 2020). As a result, post-2008 deforestation has continued to offset restoration gains, leading to a near-stagnant APP deficit between 2008 and 2021.

Taken together, our results highlight the need for systematic, large-scale monitoring of LPVN implementation by state environmental agencies. Applying standardized, spatially explicit methodologies at basin and state scales can serve as an early-warning system to identify critical regions where new deforestation is offsetting restoration efforts, to track progress toward legal compliance, and to distinguish areas where restoration obligations, compensation opportunities, or preventive conservation actions should be prioritized. Such monitoring frameworks can support more strategic allocation of enforcement, technical assistance, and incentive-based instruments, while increasing transparency and accountability in LPVN implementation. By linking legal compliance metrics to spatial patterns of land-use change, states can move beyond reactive enforcement and toward proactive, evidence-based strategies to prevent new deforestation and enhance the ecological effectiveness of restoration and compensation policies.

Despite these insights, some limitations of this study should be acknowledged. First, our assessment of restoration progress relied exclusively on remote sensing–derived land-cover maps, which allow the identification of forested versus non-forested areas but do not provide information on vegetation condition or successional stage. As a result, areas classified as having progressed toward compliance reflect the presence of detectable forest cover following classification as deforested in a previous year.

Additionally, despite potential spatial overlap between APP and RL areas, this study assessed their deficits separately for each legal category. Some areas classified as consolidated within RL surpluses may overlap with APP zones that remain subject to mandatory restoration, potentially leading to underestimation of restoration obligations when APPs are assessed in isolation. This highlights the need for integrated analytical approaches that jointly consider APP and RL requirements to better capture the full scope of legal and ecological liabilities under the LPVN.

## Conclusions

This study provides a fine-scale evaluation of forest-law implementation at the property level over time in a representative region of the Brazilian Legal Amazon, characterized by a landscape roughly divided between protected areas and private lands. It demonstrates that the effectiveness of forest protection laws depends not only on the legal framework itself, but fundamentally on their capacity to prevent new deforestation while enabling feasible restoration pathways. In the Itacaiúnas River Basin, the persistence of forest deficits demonstrates that restoration efforts have been largely offset by continued forest loss, revealing a structural mismatch between legal expectations and implementation capacity.

Our findings show that compensation mechanisms embedded in the LPVN offer a technically viable solution to resolve a substantial share of existing RL deficits within the basin itself. However, when compensation relies predominantly on forest areas that are already legally protected the ecological additionality of these mechanisms becomes limited. At the same time, such arrangements may play a critical role in stabilizing land use and supporting vulnerable populations, highlighting a trade-off between ecological gains and social outcomes that deserves policy attention.

The stagnation of APP restoration further indicates that legal obligations alone are insufficient to ensure recovery of environmentally sensitive areas. Without effective enforcement, monitoring, and incentives aligned with local land-use realities, restoration targets remain largely symbolic.

Ultimately, the analysis of results by land tenure category underscore a broader lesson highlighted by multiple studies: forest policy in the Amazon will only succeed when compliance becomes economically viable, socially legitimate, and institutionally enforceable. This requires integrating forest restoration with rural development, strengthening land tenure security, and redesigning compensation mechanisms to ensure genuine ecological additionality and equitable outcomes.

## Supplementary information

Supplementary material

## Data Availability

The research data supporting the findings of this study are available from the corresponding authors upon request.
