# Supplementary material for: Slow Progress Under Brazil’s Native Vegetation Protection Law in the Southeastern Amazon
Source: Environ Manage. 2026 Apr 21;76(5):170. doi: 10.1007/s00267-026-02454-9 (PMC13099751; doi:10.1007/s00267-026-02454-9)
Supplement: Supplementary file 1 — Supplementary material [file 267_2026_2454_MOESM1_ESM.docx]

# Suplementar Material

## Land use comparison with MapBiomas

We compared the BHRI land cover classification proposed here with the mapping from MapBiomas project (Collection 7). To perform the comparison, we randomly sampled 1000 pixels from our forest area classification (ITV) and compared these points with the MapBiomas map. Subsequently, we verified that 180 points presented different results and performed an additional validation for these points using high-resolution images from Google Earth (GE) for the BHRI region, available for 2021. We used visual interpretation of the GE images to classify the points into forest and deforestation. Using the reference dataset (high-resolution images), we calculated the percentage of points mapped to each category.

We found 82% (n = 820) agreement between MapBiomas and the ITV classification and 18% (n = 180) disagreement. However, we were unable to perform 41-point validation because of the lack of images available for 2021. Thus, 139 points were left for validation. Of these, 58% were classified as forest (80 points) by visual interpretation using GE imagery. Through the visual interpretation of high-resolution images from 2021, we were also able to verify that most of the divergent points between ITV classification and MapBiomas were found in the forest edge area or at some stage of regeneration.

It should be noted that the collection of images used by MapBiomas is different from those used by ITV. The first classifies Landsat images, with a spatial resolution of 30 meters, and the second the Sentinel-2 images, with a spatial resolution of 10 meters. In addition to spatial resolution, differences are observed between the mapped area (scale), the spectral sensitivity and radiometric quality of the sensors, the number of bands and indices used and the classification techniques adopted.

MapBiomas uses a pixel-oriented classification approach through the automatic Random Forest classifier that is based on machine learning and is fed by samples of defined classes. ITV uses an object-oriented classification approach (GEOBIA) that segments an image and groups pixels by their similar characteristics. Both techniques used to classify images present good results, with GEOBIA usually obtaining better results when used on images with better spatial resolution.

The differences between the mappings are acceptable considering the points mentioned, mainly the classification technique. The choice of image collection by MapBiomas results from the need to map the entire extent of the Brazilian territory while ITV needs to understand the processes of change in land cover and use in a regional river basin, that is, the mapping scales are different. For a regional study, ITV chose to classify land cover in the Itacaiúnas river basin using images with better spatial definition at the expense of the availability of spectral bands. In this sense, the use of GEOBIA was the choice to classify the Sentinel-2 images. It is noteworthy that the mapping prepared by ITV achieved agreement with the classification above 94% in the years classified with Landsat images (1984, 1994, 2004, 2008 and 2013) and Sentinel-2 (2017 and 2021) (Souza Filho et al. ,2018). The kappa index values, to evaluate real agreement by eliminating random agreement, were greater than 0.9. Allocation and quantity errors were less than 3.5% and 2% respectively. User and producer accuracies were greater than 80%.

In addition, a classification approach based on GEOBIA has many advantages over pixel-based classification (Liu and Xia, 2010; Souza-Filho et al., 2018). The GEOBIA approach enables the use of additional information (brightness, shape, neighborhood, and distance), that exceeds the digital pixel value (Blaschke et al., 2014; Burnett and Blaschke, 2003). Geographic objects formed from the grouping of pixels with this complementary information allow a more accurate representation of the elements in the real world. This explains the larger forest area mapped in our analysis (SM1). The classification approach used here allows for the capture of forest areas that may be lost in a pixel-based classification approach.


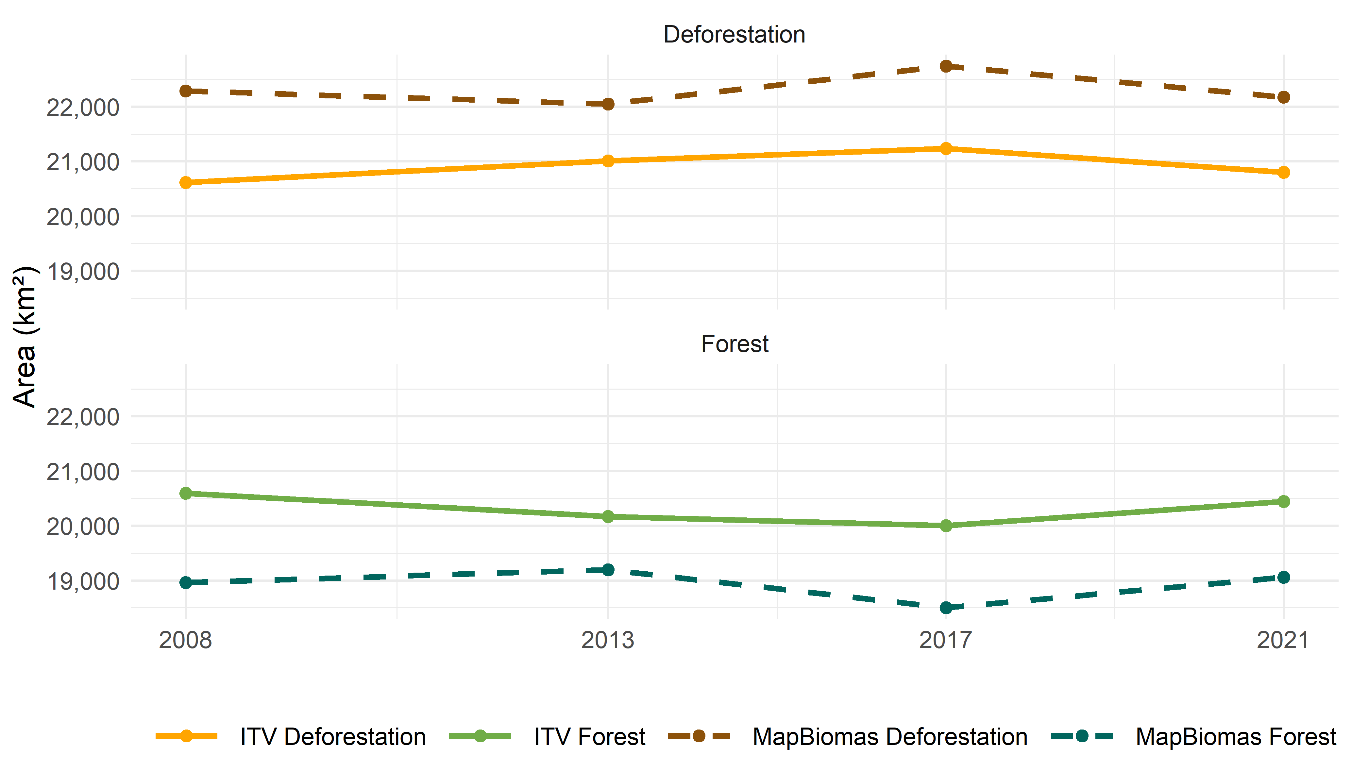
 **SM1.** Validation of the land use and cover classification used (Sentinel-2 / 10 m) with the classification of the Annual Mapping Project of Land Use and Coverage in Brazil (MapBiomas - Landsat / 30 m) for the Itacaiúnas River basin, located in the state of Pará, southeastern Brazilian Amazon.

## Analyses of RL by municipalities

Three municipalities in the basin account for 86% of the deforested surplus: Marabá (63% or 117 km²), Água Azul do Norte (16% or 31 km²), and Parauapebas (7% or 13 km²). Moreover, 70% of the surplus available for compensation is in the municipalities of Marabá (49%, or 1,830 km²), Parauapebas (11%, or 411 km²), and Eldorado dos Carajás (10%, or 367 km²) (Figure 5).


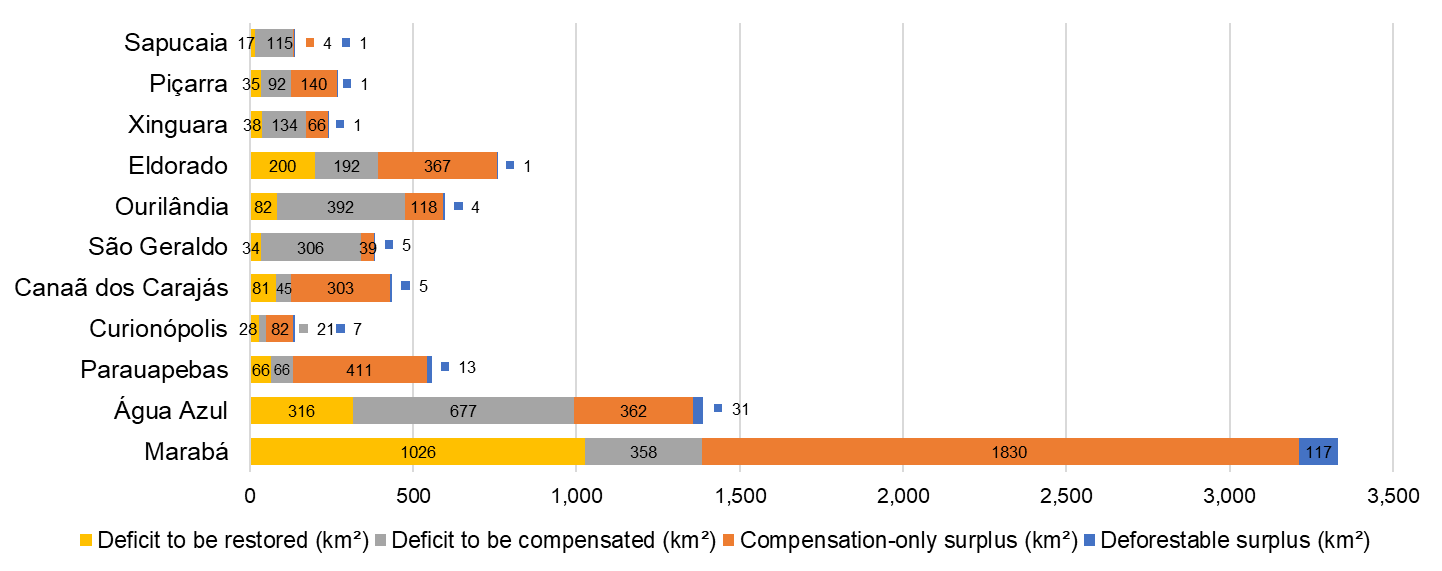


**SM2**. Deforestable surplus, surplus for compensation, deficit to be compensated, and deficit to be restored by municipality, according to Law Nº. 12,651 of May 25, 2012, in the Itacaiúnas Basin, located in the state of Pará, southeastern Brazilian Amazon. We considered only the area of the municipality that was included in the basin.

## Analyses of APP by municipalities

Approximately 90% of the APP’s forest area is concentrated in the five largest municipalities in the basin: Marabá (1,199 km² or 38%), Parauapebas (824 km² or 26%), Água Azul do Norte (418 km² or 13%), Canaã dos Carajás (239 km² or 8%), and Curionópolis (125 km² or 4%). Approximately 80% of the deficits to be recovered were distributed among the municipalities of Água Azul do Norte (323 km² or 25%), Marabá (327 km² or 26%), Eldorado dos Carajás (121 km² or 10%), Curionópolis (123 km² or 10%), and Canaã dos Carajás (98 km² or 8%). Most of the consolidated APP areas were concentrated in five municipalities: 24% in Marabá (203 km²), 19% in Água Azul do Norte (160 km²), 17% in Eldorado dos Carajás (142 km²), 9% in Canaã in Carajás (77 km²), and 9% in São Geraldo do Araguaia (72 km²) (SM 3).


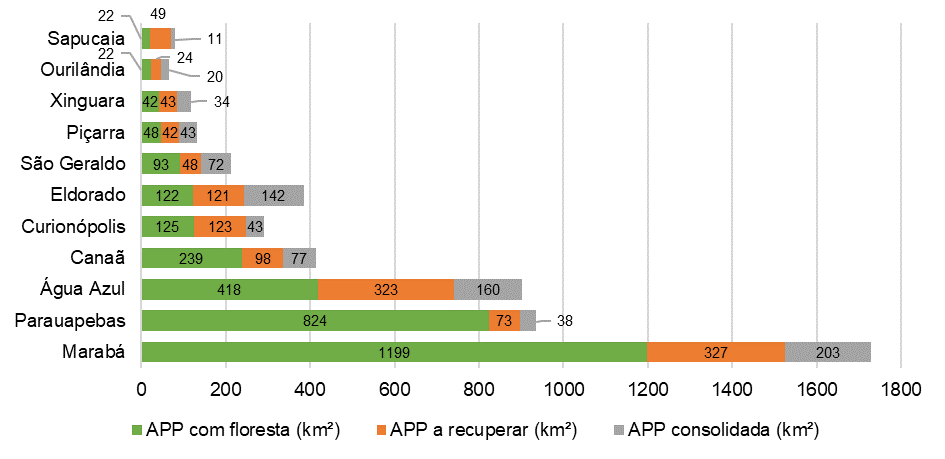


**SM3**. According to Law Nº. 12,651 of May 25, 2012, APP with forest, APP to recover, and APP consolidated by municipalities in the hydrographic basin of the Itacaiúnas River, located in the state of Pará, southeastern Brazilian Amazon.

**References**

Blaschke T, Hay GJ, Kelly M, Lang S, Hofmann P, Addink E, Queiroz Feitosa R, van der Meer F, van der Werff H, van Coillie F, Tiede D (2014) Geographic Object-Based Image Analysis—Towards a new paradigm. ISPRS J Photogramm Remote Sens 87:180–191. https://doi.org/10.1016/j.isprsjprs.2013.09.014.

Burnett C, Blaschke T (2003) A multi-scale segmentation/object relationship modelling methodology for landscape analysis. Ecol Model 168(3):233–249. https://doi.org/10.1016/S0304-3800(03)00139-X.

Liu D, Xia F (2010) Assessing object-based classification: Advantages and limitations. Remote Sens Lett 1(4):187–194. https://doi.org/10.1080/01431161003743173 .

Souza-Filho, P. W. M., Nascimento, W. R., Santos, D. C., Weber, E. J., Silva, R. O., & Siqueira, J. O. (2018). A GEOBIA approach for multitemporal land-cover and land-use change analysis in a tropical watershed in the southeastern Amazon. Remote Sensing, 10(11), 1683. https://doi.org/10.3390/rs10111683
